# PERCEIVED AND ACTUAL BMI IN AGING ADULTS: A SCOPING REVIEW

**DOI:** 10.1093/geroni/igae098.3400

**Published:** 2024-12-31

**Authors:** Sarah Hubner, Julie Blaskewicz Boron

**Affiliations:** Oregon Health Authority, Public Health Division, Health Promotion and Chronic Disease Prevention, Camas, Washington, United States; University of Nebraska Omaha, Omaha, Nebraska, United States

## Abstract

Sustaining a healthy weight across the lifespan is an important aspect of disease prevention and quality of life in aging. Still, approximately 77% of US adults deviate from ideal weight recommendations, contributing to disease and disability. Improving lifestyles is critical to reducing this chronic disease burden. Self-perception of weight status (perceived-BMI) and its accuracy (versus actual-BMI) may influence behavior through goal setting and motivation; however, this relationship remains understudied in aging adults. Examining BMI dissonance and its broader context within the aging population may contribute to better understanding how self-perception shapes behavior and wellness. The current study contributes to this goal by assessing the field via scoping review. Electronic database searches were carried out in: PubMed, APA PsycInfo, Sports Medicine & Education Index. Peer reviewed white literature published in English from database inception to May 2024 were included in the search. Eligible research was related to aging adults’ body form and included primary measures for perceived- and actual-BMI. Populations were community-dwelling adults, aged 45+. The search revealed 4160 titles. After title, abstract, and full text screen, 13 articles were selected for inclusion. Evaluation of the selected studies showed that diverse aspects of perceived-BMI were described, including diagnostic accuracy and alignment with actual-BMI, association with health literacy, behavioral impacts, and correlations to lifestyle change strategies. These synthesized results may contribute to understanding how self-perception shapes behavior and wellness; discussion will include how this review can inform interventions to promote healthier lifestyles and reduce chronic disease burden across the adult lifespan.

